# Kinesin-5 motors are required for organization of spindle microtubules in *Silvetia compressa *zygotes

**DOI:** 10.1186/1471-2229-6-19

**Published:** 2006-08-31

**Authors:** Nick T Peters, Darryl L Kropf

**Affiliations:** 1 Department of Biology, University of Utah, Salt Lake City, UT 84112, USA

## Abstract

**Background:**

Monastrol, a chemical inhibitor specific to the Kinesin-5 family of motor proteins, was used to examine the functional roles of Kinesin-5 proteins during the first, asymmetric cell division cycle in the brown alga *Silvetia compressa*.

**Results:**

Monastrol treatment had no effect on developing zygotes prior to entry into mitosis. After mitosis entry, monastrol treatment led to formation of monasters and cell cycle arrest in a dose dependent fashion. These findings indicate that Kinesin-5 motors maintain spindle bipolarity, and are consistent with reports in animal cells. At low drug concentrations that permitted cell division, spindle position was highly displaced from normal, resulting in abnormal division planes. Strikingly, application of monastrol also led to formation of numerous cytasters throughout the cytoplasm and multipolar spindles, uncovering a novel effect of monastrol treatment not observed in animal cells.

**Conclusion:**

We postulate that monastrol treatment causes spindle poles to break apart forming cytasters, some of which capture chromosomes and become supernumerary spindle poles. Thus, in addition to maintaining spindle bipolarity, Kinesin-5 members in *S. compressa *likely organize microtubules at spindle poles. To our knowledge, this is the first functional characterization of the Kinesin-5 family in stramenopiles.

## Background

Fucoid algae are model organisms uniquely suited for studies investigating cellular polarity and asymmetric division during development. The fucoid marine brown alga *Silvetia compressa*, a member of the stramenopile lineage, displays oogamous fertilization in which a large sessile egg is fertilized by a small motile sperm [1]. The eggs and zygotes are large, about 100 μm in diameter, facilitating physiological and microscopic investigations [2]. Large numbers of zygotes, released into the surrounding seawater, can be easily collected for experimentation [3]. Zygotes develop synchronously during the first division cycle, completed about 24 h after fertilization (AF) with an invariant, asymmetric cell division [4]. From fertilization through cell plate deposition, a dynamic microtubule (MT) network is utilized for multiple cellular processes including migration of the male pronucleus, separation of the centrosomes around the nuclear envelope, nuclear positioning and rotation, formation and maintenance of a properly positioned mitotic spindle, and formation of a cytokinetic plane that bisects the spindle [5]. Numerous investigations of cytoskeletal proteins in *S. compressa *have provided a framework for more detailed studies examining the function of cytoskeletal associated proteins, including molecular motors [4,6,7]. This study examines the Kinesin-5 family of motors during early *S. compressa *development.

The kinesin superfamily proteins (KIFs) are a diverse and evolutionarily conserved group of molecular motors present in metazoans, plants, fungi, and protozoans [8]. Kinesins possess a ~360 amino acid residue globular "motor" domain that hydrolyzes ATP for movement along MTs [8]. Kinesins travel towards the plus or minus end of MTs, or perform more structural roles in MT organization and stability [9]. The "tail" domains of kinesins are much less conserved and can mediate formation of higher order structures with fellow kinesins or attachment of cargos to be transported along MTs [8]. Recent work has categorized kinesin family members into 14 different classes which function in a multitude of cellular processes [9]. While human and fly kinesins have recently been systematically examined to determine their roles in cellular and developmental processes [10,11], the functional roles of kinesins in stramenopiles have not been investigated.

Monastrol and a chemical analogue *S*-trityl-L-cysteine (STLC) are small, cell-permeable inhibitors of the Kinesin-5 family of plus-end directed motors [12]. Kinesin-5 motors are bipolar homotetramers with two motor domains at each end, separated by a stalk/tail region [13]. Kinesin-5 motors are localized to the nucleus in animal cells until nuclear envelope breakdown at the onset of mitosis [14]. Inhibition of this motor has severe effects in mitosis but little or no effect in interphase [15]. During mitosis, Kinesin-5 motors function near the spindle midzone to maintain pole to pole distance. Motor domains attach to MTs from opposite poles and translocate towards the plus ends, thereby pushing spindle poles apart [16]. Additionally, Kinesin-5 proteins have been observed at spindle poles, though their function at that location is unclear [16,17]. Monastrol acts by specifically binding to and inhibiting the motor domain of Kinesin-5 proteins, thus impeding processive movement, while not affecting MT binding interactions [18]. Inhibition of processive movement of motors at the spindle midzone leads to formation of "monaster" spindles during metaphase. Monasters are defined by collapse of the spindle poles to a single focus, with captured chromosomes at the distil, plus ends [18].

To begin addressing the functional roles of kinesins during development in *S. compressa*, we examined the effects of monastrol treatment on the first asymmetric cell division cycle. We found that monastrol treatment induced formation of monasters upon entry into mitosis, confirming previous findings with animal systems [18]. Novel structures were also observed following drug treatment; multiple cytasters were observed throughout the cytoplasm and many cells formed multipolar spindles. There was a strong correlation between the presence of cytasters and multipolar spindles. These findings suggest that monastrol induces spindle pole breakup at mitosis entry, resulting in formation of cytasters, some of which become supernumerary spindle poles. Thus, Kinesin-5 members in *S. compressa*, and perhaps in other stramenopiles, appear to function not only at the spindle midzone to generate bipolarity, but also at spindle poles to maintain pole integrity.

## Results

### Monastrol or STLC treatment induced formation of monasters and multipolar spindles

**Figure 1:**
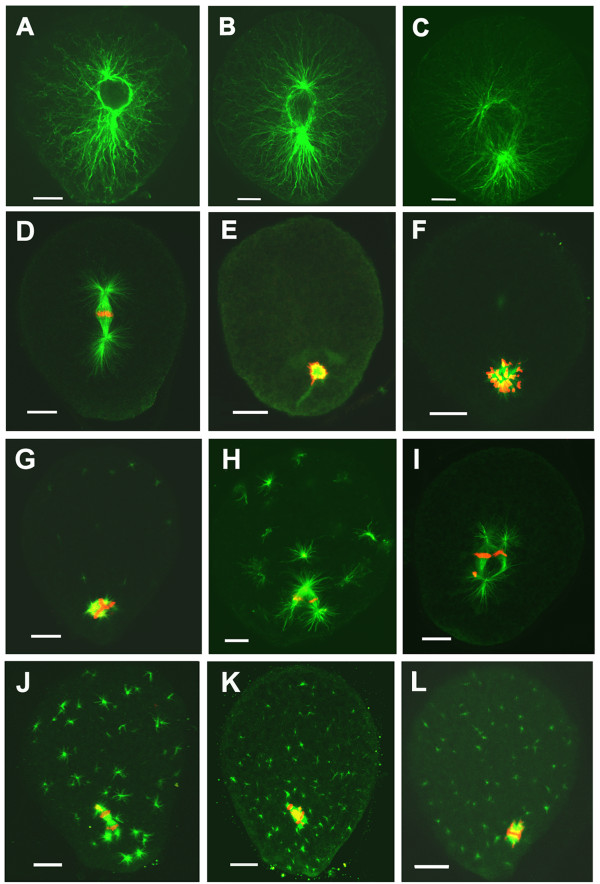
Monastrol and STLC treatment of *S. compressa *zygotes. (A-C) Zygotes were not sensitive to monastrol treatment during interphase (16 h AF); (A) 0.2% DMSO (B) 25 μM monastrol, (C) 50 μM monastrol. Note that the interphase zygotes in B and C exhibit elongated nuclei, indicating that they developed slightly faster than the control zygote in A. However, differences in developmental timing between treatments were not routinely observed. (D-F) Formation of monasters in mitosis (24 h AF); (D) 0.2% DMSO, (E) 25 μM monastrol, (F) 5 μM STLC. (G-I) Multipolar spindles (24 h AF); (G) 25 μM monastrol, (H) 50 μM monastrol, (I) 0.5 μM STLC. (J-L) Cytaster formation; (J) 25 μM monastrol (24 h AF), (K) 50 μM monastrol (24 h AF), (L) 100 μM monastrol (48 h AF). Cytasters were observed scattered throughout the cytoplasm. Hours in parentheses indicate times at which zygotes were fixed. MTs are depicted in green, condensed chromatin is depicted in red, and overlap between MTs and condensed chromatin is depicted in yellow. Median optical sections, 10–20 μm in total thickness, are shown. Scale bars equal 10 μm.

To determine the functions of Kinesin-5 motors in brown algal cells, monastrol (25 μM – 100 μM) and a more potent chemical analogue, STLC (0.5 μM – 10 μM), were applied to *S. compressa *zygotes at 6 h AF. Cells were subsequently fixed at 16, 24, and 48 h AF, and fluorescently labeled with antibodies to observe MTs and condensed chromatin by confocal microscopy (see Materials and Methods). Monastrol had no effect on development or MT arrays of zygotes observed 16 h AF, prior to entry into mitosis. Treated zygotes germinated in accordance with an orienting light vector and had extensive MT arrays emanating from centrosomes on opposite sides of the nucleus (Fig. 1A–C). This finding suggests nuclear sequestration of Kinesin-5 motors during interphase, as has been reported in animal cells [10,19,20].

Normal metaphase spindles are characterized by two distinct spindle poles, consisting of centrioles and a cloud of hundreds of pericentriolar matrix proteins [21], spindle MTs and condensed chromatin at a metaphase plate (Fig. 1D). Treatment with monastrol severely disrupted spindle formation and resulted in monaster formation in some zygotes (Fig. 1E–F). Monasters, astral arrays of MTs with condensed chromatin near the periphery of the array, were similar to those observed in monastrol-treated or Kinesin-5-depleted animal cells [10,19,20]. Intense labeling of MTs in monasters is consistent with large numbers of short MTs in close proximity [16]. These results indicate that monastrol and STLC specifically inhibit brown algal Kinesin-5 motors that are critical for bipolar spindle assembly and maintenance. Although both drug treatments had similar effects on zygotes, monastrol treatment produced the most consistent results and was therefore the main focus for subsequent work.

**Figure 2:**
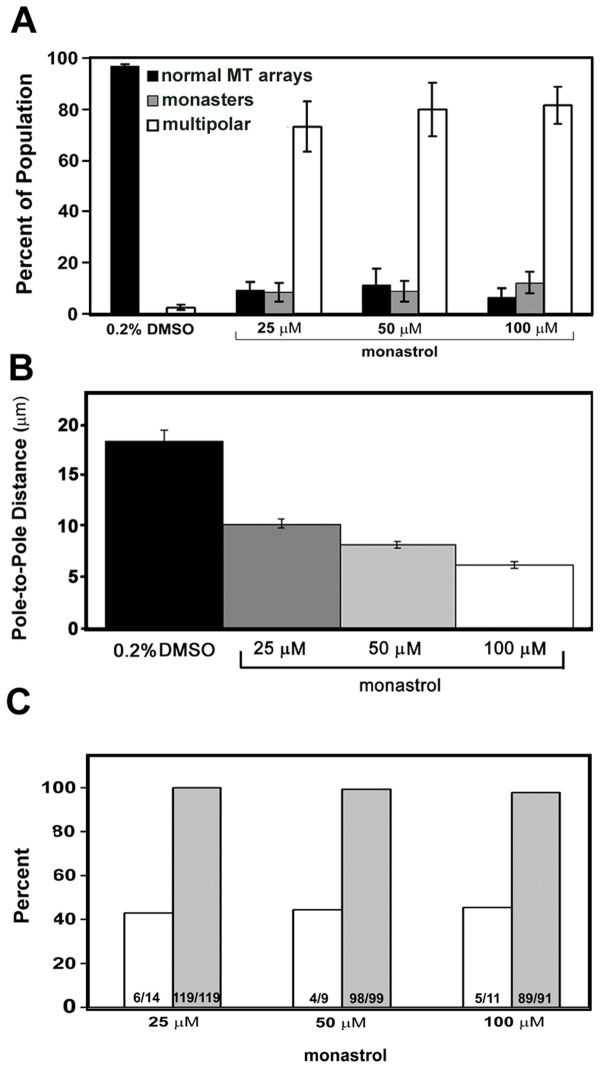
Monastrol or STLC treatment induced formation of multipolar spindles with reduced pole-to-pole distance at 24 h AF. (A) Incidence of monasters, multipolar spindles and normal MT arrays with increasing monastrol concentration. Zygotes with normal MT arrays had progressed beyond metaphase at 24 h AF; most were in telophase. (B) Pole-to-pole distance. For multipolar spindles, only the greatest distance between any two spindle poles sharing captured chromosomes was measured. Pole-to-pole distance was significantly (p < 0.001, Student's t test) shorter in all drug treatments compared to controls, and all treatments were significantly different from each other. (C) Multipolar spindles were associated with cytasters. Open bars; percent of the cells with monasters that also had cytasters. Shaded bars; percent of cells with multipolar spindles that also had cytasters.

Surprisingly, most treated cells formed multipolar spindles rather than monasters at entry into mitosis. Multipolar spindles have not been observed in other organisms treated with monastrol. The structure of multipolar spindles was quite variable, ranging from three spindle poles arranged linearly with chromosomes between poles to three or more poles in various spatial arrangements with captured chromosomes at metaphase plates (Fig. 1G–I). Chromosomes captured by a single spindle pole were also observed, but "lost" chromosomes unassociated with spindle MTs were not found. Multipolar spindles were the predominant mitotic structure in treated cells, and were observed in 70–80% of the population (Fig. 2A). The remaining 20–30% of the population had normal MT arrays or monasters in roughly equal proportions. Regardless of spindle morphology, pole-to-pole separation was reduced in a dose dependent manner by monastrol treatment (Fig. 2B), consistent with the postulated role of Kinesin-5 in generating spindle bipolarity.

### Cytasters were present in most treated cells

Most treated cells observed after entry into mitosis possessed cytasters (Fig. 1H, J–L). Cytasters are supernumerary MT organizing centers (MTOCs) [22]. These structures are reminiscent of the star-shaped MT-based structures observed in the *Fucus serratus *cell cortex by Corellou *et al*. [7]; however, in our study cytasters were only observed after monastrol treatment and were distributed throughout the cytoplasm. MTs in cytasters were shorter than centrosomal MTs of untreated controls. Cytasters were present in nearly all zygotes that had multipolar spindles, while less than half of zygotes with monasters displayed cytasters (Fig. 2C). The strong correlation between multipolar spindles and cytasters suggests that cytasters may function as supernumerary spindle poles. Like multipolar spindles, cytasters have not been observed in other cell types or organisms treated with monastrol.

### Monastrol treatment delays or inhibits cell division

**Figure 3:**
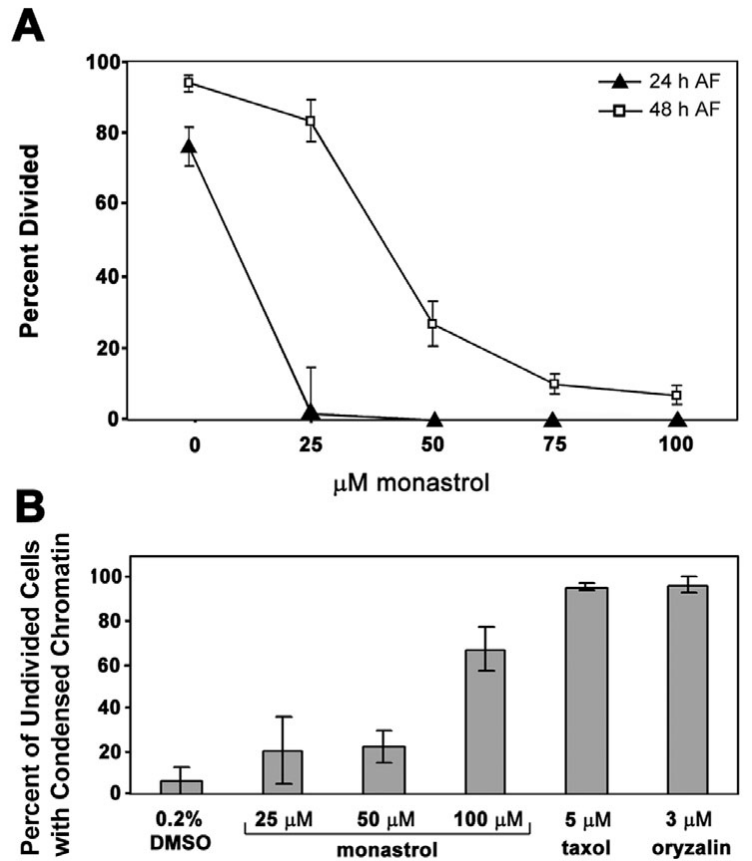
Monastrol treatment led to cell cycle delay at low concentrations and cell cycle arrest at high concentrations. (A) Filled triangles depict cell plate formation at 24 h AF, and open squares depict cell plate formation at 48 h AF. (B) Chromatin condensation in undivided cells at 48 h AF assayed by labeling with antibody to phosphorylated histone H3. Bars are standard errors.

Monastrol delayed cell division at low drug concentrations and inhibited cell division at higher concentrations (Fig. 3A). While cell division was significantly reduced by all drug treatments at 24 h AF, most zygotes treated with 25 μM monastrol had divided by 48 h AF. By contrast, treatment with higher drug concentrations (50 μM – 100 μM) resulted in sustained inhibition of cell division in a dose dependent fashion. The monastrol concentrations leading to cell cycle arrest are consistent with IC_50 _values reported for inhibition of ATP binding by a racemic mixture of monastrol [18], providing further evidence that monastrol specifically targets *S. compressa *Kinesin-5. Many undivided cells appeared to be arrested in first mitosis as judged by prolonged phosphorylation of histone H3. Histone H3 is phosphorylated from late prophase until global dephosphorylation during anaphase [23]. At 48 h AF, the percent of undivided zygotes with persistent histone H3 phosphorylation was concentration dependent in monastrol (Fig. 3B). In 100 μM monastrol, 66.5 ± 10.1 percent of the undivided cells exhibited condensed chromatin. These zygotes had likely arrested at the spindle assembly checkpoint of the first cell cycle [24]. Indeed, when the spindle assembly checkpoint was activated by MT stabilization using paclitaxel or MT depolymerization using oryzalin, nearly all zygotes exhibited prolonged histone H3 phosphorylation (Fig. 3B).

### Spindle and division plane alignment

Metaphase spindles in untreated cells were reasonably well aligned with the growth axis and resided near the center of the zygote (Fig. 1D). Interestingly, aberrant spindles in monastrol-treated zygotes were often displaced toward the rhizoid (Fig. 1E–L). Condensed chromatin was also incorrectly positioned following MT depolymerization by oryzalin or MT stabilization by paclitaxel, as has been previously reported [25]. However, preferential displacement toward the rhizoid was not observed in paclitaxel or oryzalin; chromatin position instead appeared more random (data not shown). Together these findings support the hypothesis that MTs are crucial for spindle positioning [26,27].

**Figure 4:**
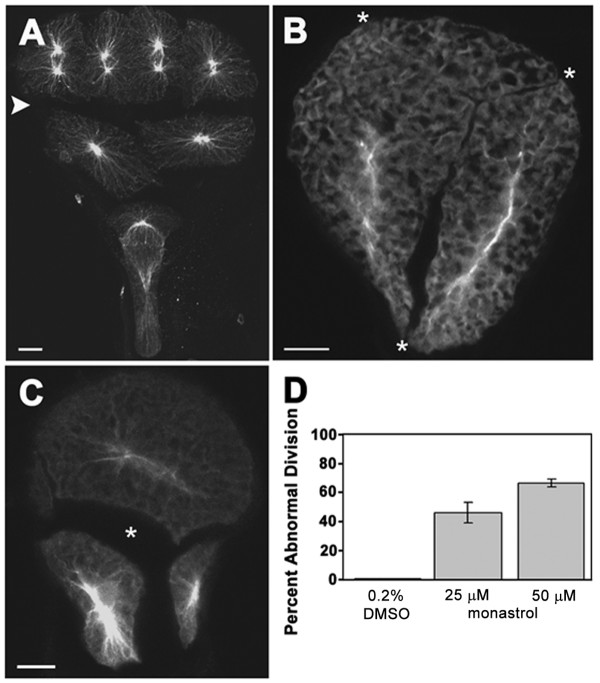
Monastrol concentrations permissive for cell division led to aberrant cytokinesis assayed at 48 h AF. (A) 0.2% DMSO. By 48 h AF, control zygotes had divided several times in a stereotypical pattern. Arrowhead indicates first division plane. (B-C) 25 μM monastrol. Asterisks mark aberrant division planes. (D) Percent of divided zygotes with abnormal first cytokinesis, as assessed by >30 degree deviance from perpendicular to the long axis of the cell, or by curved cell plates. Standard error bars shown. Scale bars equal 10 μm.

*S. compressa *zygotes determine division plane based on spindle position [26], so monastrol treatment was predicted to alter division patterns. The first asymmetric division in untreated zygotes bisected the spindle and therefore was transverse to the growth axis (arrowhead, Fig. 4A). Since few zygotes treated with 100 μM monastrol divided, we focused on lower drug concentrations. As expected, aberrant division planes (assessed as >30 degrees from perpendicular to the long axis of the cell) were observed in cells treated with 25 μM or 50 μM monastrol (Fig. 4B). In addition, curved planes of division were also observed (Fig. 4C). The percent of cells with abnormal division planes increased with monastrol concentration; at 50 μM approximately two thirds of divided cells displayed aberrant division planes (Fig. 4C).

### MT disruption slows tip growth

**Figure 5:**
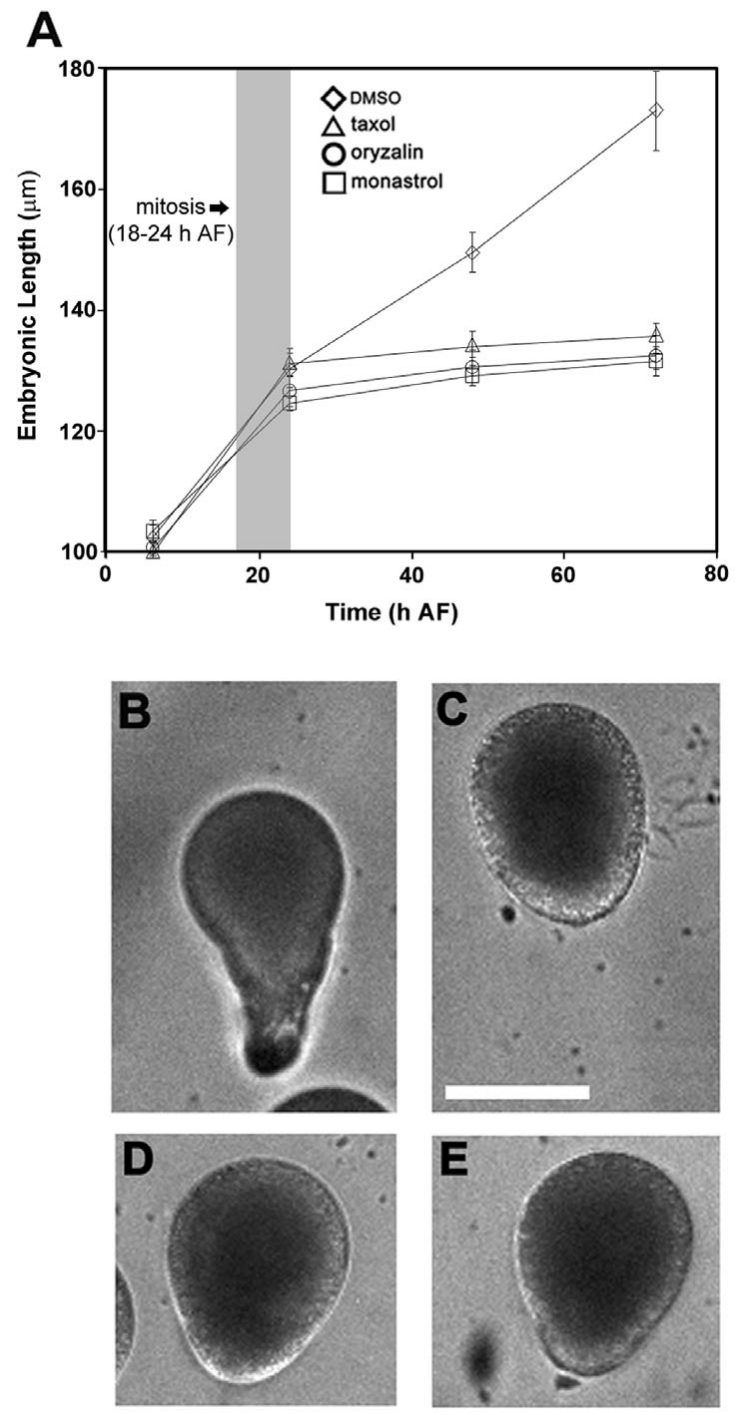
Monastrol, paclitaxel, and oryzalin severely reduce tip growth at first mitosis. (A) Embryonic growth over a 72 h period. Data are from one representative experiment sampling ten undivided zygotes for each treatment. Diamonds, 0.2% DMSO; triangles, 5 μM paclitaxel; squares, 3 μM oryzalin; circles, 50 μM monastrol. Standard error bars are shown. (B-E) Images of treated zygotes at 72 h AF. (B) 0.2% DMSO, (C) 50 μM monastrol (D) 5 μM paclitaxel, (E) 3 μM oryzalin. Scale bar equals 100 μm.

Rhizoids of zygotes treated with paclitaxel, oryzalin or monastrol appeared shorter and broader than control rhizoids (Fig. 5B–E). We therefore assayed tip growth by measuring the length of the embryonic axis over a three-day period. None of the pharmacological agents affected rhizoid growth prior to mitosis; zygotes elongated at ~1 μm/h regardless of treatment (Fig. 5A). Although control embryos continued to elongate at ~1 μm/h after first division, tip elongation was severely reduced in treated zygotes following entry into first mitosis. This growth reduction was probably not due to MT disruption since treated zygotes grew normally prior to mitosis, nor was it likely caused by a cell volume checkpoint. Fucoid zygotes treated with aphidicolin activate an S-phase cell cycle checkpoint that blocks nuclear division and cytokinesis, but permits continued rhizoid tip growth [24]. This results in very large, elongated zygotes that are undivided, suggesting that zygotes do not possess a volume checkpoint. Instead, the abrupt reduction in growth rate we observed was probably caused by arrest in M phase, perhaps via activation of the spindle assembly checkpoint [28].

## Discussion

Although assembly of a mitotic spindle is not well understood, the process requires dynamic MTs, plus-end and minus-end directed MT motors, and a plethora of accessory proteins, including static crosslinking molecules [29]. Kinesin-5 motors localize within the nucleus until nuclear envelope breakdown (NEB) [14] when they are released into the cytoplasm and accumulate at the midzone and poles of the forming spindle. They are required for maintenance of bipolar spindle integrity and poleward flux of MTs [30]. In the midzone, Kinesin-5 motors interact with MTs from opposite poles and move at ~20 nm s^-1 ^toward MT plus-ends, effectively pushing spindle poles apart [16]. Inhibition of Kinesin-5 function by gene knockout, RNAi-mediated depletion, or treatment with pharmacological agents results in monaster formation during spindle assembly [10,11,19]. For example, mutations in *Drosophila *KLP61F, a Kinesin-5 ortholog, have been shown to disrupt maintenance of spindle pole separation [31]. Kinesin-5 proteins act in concert with other spindle associated proteins to maintain proper spindle length. For example, increasing the levels of NuMA, a MT crosslinking protein, restores spindle bipolarity in mitotic extracts lacking Kinesin-5 activity [32]. In sum, animal Kinesin-5 motors function with other mitotic proteins to generate and maintain pole-to-pole separation by pushing apart antiparallel MTs at the spindle midzone.

The roles of kinesins in the stramenopile lineage have not been functionally investigated. We examined the roles of Kinesin-5 motors during the first cell division cycle in *S. compressa *by employing Kinesin-5-specific inhibitors to brown algal zygotes. *S. compressa *zygotes treated with monastrol (or STLC) appeared normal and had normal MT arrays in interphase of the first cell cycle, consistent with nuclear localization prior to NEB. Unfortunately, we were unable to confirm nuclear localization since available antibodies to animal Kinesin-5-proteins did not label fucoid zygotes. Importantly, monasters were formed upon entry into mitosis. These findings are similar to reports in animals [10,11,19] and indicate that monastrol binds and inhibits Kinesin-5 motors in *S. compressa*. However, monastrol had additional effects on zygotes not observed in animal cells; in addition to monasters, multipolar spindles and cytasters were formed at mitotic entry.

Multipolar spindles were formed at a much higher frequency than monasters, indicating that spindle poles do not fully collapse when Kinesin-5 motors are inhibited. This is unrelated to monastrol dosage since increasing concentration did not significantly increase monaster frequency. Instead, zygotes probably possess other mechanisms that work in concert with Kinesin-5 to maintain pole separation. There may be MT-based motors with overlapping function or MT crosslinking proteins, such as NuMA, that continue to function in the presence of monastrol, maintaining some pole separation. Centrosome position at the onset of mitosis may also contribute to the relatively low monaster frequency. Brown algal centrosomes are fully separated on the nuclear envelope prior to entry into mitosis, residing about 15 μm apart [33]. Animal centrosomes, however, separate concurrent with NEB so spindle poles are still close together when Kinesin-5 motors become active [34]. The greater separation of algal spindle poles may reduce the likelihood of complete spindle collapse to a monaster during drug treatment.

The presence of multipolar spindles also implies that supernumerary spindle poles are formed at entry into mitosis, and the extra poles may be derived from cytasters. Although the composition of fucoid cytasters is unknown, they are distinct small astral-like radial bursts of MTs. These are commonly associated with centrin labeling in other systems, and are likely MTOCs [22]. The observation that nearly all zygotes with multipolar spindles additionally displayed cytasters, while cytasters were only present in about half of cells with monasters, suggests a causal a link between cytasters and supernumerary spindle poles. We speculate that monastrol treatment leads to spindle pole breakup in *S. compressa *and fragments of spindle poles nucleate MTs and become cytasters. Numerous cytasters are located throughout the cytoplasm of treated cells, and occasionally one residing close to condensed chromatin captures chromosomes, thereby becoming a supernumerary spindle pole. In this model, Kinesin-5 motors must organize and maintain the integrity of spindle poles. Kinesin-5 members residing at spindle poles have been postulated to bundle long MTs [35], sort numerous short MTs in the cloud of spindle pole proteins [16], and/or mediate attachment to a hypothesized scaffold-like spindle matrix [35]. In fucoid zygotes, one or more of these putative functions may be needed to hold pole components together.

Interestingly, the vast majority of zygotes treated with monastrol had aberrant spindles that were displaced from a central cellular position toward the rhizoid. Proper alignment of the mitotic spindle in brown algae has been shown to be a MT-dependent process [27], and we observed that condensed chromatin was displaced in zygotes treated with paclitaxel or oryzalin (data not shown). MTs that position the fucoid spindle are thought to do so by interacting with the cell cortex before and after metaphase [26]. Perhaps the dramatically prolonged metaphase during monastrol treatment permits unregulated spindle movement, resulting in aberrant spindle position. Even so, it is not clear why the spindles preferentially drift in the rhizoid direction.

Cytokinesis bisects the spindle in fucoid zygotes [27], so it was not surprising to find abnormal divisions following monastrol treatment. The preponderance of multipolar spindles likely resulted in many of the abnormal divisions. Some zygotes with multipolar spindles must still progress through mitosis because the vast majority of cells treated with 25 μM monastrol display multipolar spindles at 24 h AF but completed cell division by 48 h AF. The spindle assembly checkpoint apparently does not monitor spindle pole number. Likewise, cytokinesis proceeds in sea urchin zygotes and in vertebrate somatic cells possessing supernumerary spindle poles [36]. Since it is unlikely that monaster spindles could achieve equivalent kinetochore tension on chromosomes, these zygotes probably remained arrested in metaphase and failed to divide. Therefore, most abnormal division planes in monastrol-treated *S. compressa *zygotes are likely due to multipolar or abnormally positioned spindles that achieve balanced kinetochore attachments, permitting cell cycle progression and aberrant placement of the cytokinetic plane.

Future studies will be aided by an ongoing genomic sequencing project of closely related *Ectocarpus siliculosis * [37] and by creation of an EST database for fucoid algae. This information will permit isolation of *S. compressa *Kinesin-5 sequences for use in molecular investigations and antibody production, and may provide insight into what elements, beyond MTs, are associated with cytasters.

## Conclusion

Kinesin-5 motor proteins are necessary for bipolar spindle formation during mitosis in brown algal cells, similar to what has been previously reported in animals. In addition, they appear to be involved in maintaining spindle pole integrity.

## Methods

Sexually mature fronds (receptacles) of the fucoid algae *S. compressa *were collected near Santa Cruz, California, shipped cold and stored in the dark at 4°C until use. Receptacles were potentiated by placing them under 100 μmol·m^-2^·s^-1 ^light at 16°C in artificial sea water (10 mM KCL, 0.45 M NaCl, 9 mM CaCl, 16 mM MgSO_4_, 0.040 mg chloramphenicol per ml, buffered to a pH of 8.2 with Tris base) for 1 to 3 h. Potentiated receptacles were then washed with ASW and placed in the dark for 1 h to induce gamete release. The time of fertilization was taken to be the midpoint of the dark period. Zygotes were plated onto coverslips in plastic dishes and grown in ASW with unidirectional light at 16°C until harvest. All experiments were performed in triplicate. Graphs depict data compiled from three experiments, unless otherwise noted.

Pharmacological agents were dissolved in DMSO to create stock solutions of 100 mM monastrol (AG Scientific, San Diego, Ca.), 50 mM *S*-trityl-L-cysteine (Sigma, St. Louis, Mo.), 10 mM paclitaxel (taxol, Sigma, St. Louis, Mo.) and 10 mM oryzalin. Since monastrol is light sensitive, degradation was tested by exposing 100 μM monastrol in ASW in culture dishes to unilateral light for three days. Similar levels of inhibition of cell division were observed for both fresh and light-treated monastrol, indicating no significant degradation. Drugs were applied to zygotes at various times prior to mitotic entry, yielding similar results. For all work presented here, drugs were added chronically to zygotes beginning 6 hours after fertilization (h AF). Appropriate controls were performed with DMSO at concentrations corresponding to the maximum volume of drug used. These controls showed normal development.

Pharmacological effects on tip growth were evaluated by capturing sequential images of individual embryos over three days with a coolSNAP (RS Photometrics) camera on an Olympus IMT2 microscope. Embryonic length was measured using Photoshop 7.0 (Adobe, San Jose, Ca).

For immunofluorescence microscopy of MTs and condensed chromatin, zygotes were fixed in PHEM (60 mM piperazine-N, N'-bis(2-ethanesulfonic acid), 25 mM HEPES, 10 mM EGTA, 2 mM MgCl_2_) containing 3% paraformaldehyde and 0.5% glutaraldehyde. Zygotes were processed as in Bisgrove and Kropf [26], with the following modifications. Coverslips were dipped into liquid nitrogen and removed as quickly as possible (less than 1 second) and abalone gut extract was omitted from the enzyme mixture. MTs were imaged using a monoclonal anti-α-tubulin antibody (DM1A: Sigma) with Alexa 488 goat anti-mouse secondary antibody (Invitrogen, Eugene, Or.). Condensed chromosomes were imaged using a polyclonal anti-histone H3 (pSer^10^) antibody (Calbiochem, San Diego, Ca.), followed by Alexa 546 goat anti-rabbit secondary antibody. For double labeling, primary antibodies were added simultaneously, as were secondary antibodies. Images were collected on a LSM510 (Carl Zeiss Inc., Thornwood, N.Y.) laser scanning confocal microscope with a narrow band-pass filter and Meta attachment adjustable band pass filter.

## Abbreviations

AF, after fertilization; ASW, artificial seawater; DMSO, dimethyl sulfoxide: NEB, nuclear envelope breakdown; MT, microtubule.

## Authors' contributions

NTP conducted all of the research, DLK raised the funds, and both authors contributed equally intellectually and in writing the manuscript.
